# Organic Electrochemical Transistor Aptasensor for Interleukin-6 Detection

**DOI:** 10.1021/acsami.3c12397

**Published:** 2023-12-23

**Authors:** Chiara Diacci, Bernhard Burtscher, Marcello Berto, Tero-Petri Ruoko, Samuel Lienemann, Pierpaolo Greco, Magnus Berggren, Marco Borsari, Daniel T. Simon, Carlo A. Bortolotti, Fabio Biscarini

**Affiliations:** † Laboratory of Organic Electronics, Department of Science and Technology, Linköping University, 601 74, Norrköping, Sweden; ‡ Dipartimento di Scienze della Vita, Università di Modena e Reggio Emilia, via Campi 103, 41125 Modena, Italy; § Department of Neuroscience and Rehabilitation, Università di Ferrara, Via Borsari 46, 44121 Ferrara, Italy; ∥ Center for Translational Neurophysiology of Speech and Communication, Istituto Italiano di Tecnologia, via Fossato di Mortara 17-193, 44100 Ferrara, Italy; ⊥ Dipartimento di Scienze Chimiche e Geologiche, Università di Modena e Reggio Emilia, via Campi 103, 41125 Modena, Italy

**Keywords:** Interleukin-6, Cytokine, Organic electrochemical transistor, aptasensor, Aptamer, Biosensor

## Abstract

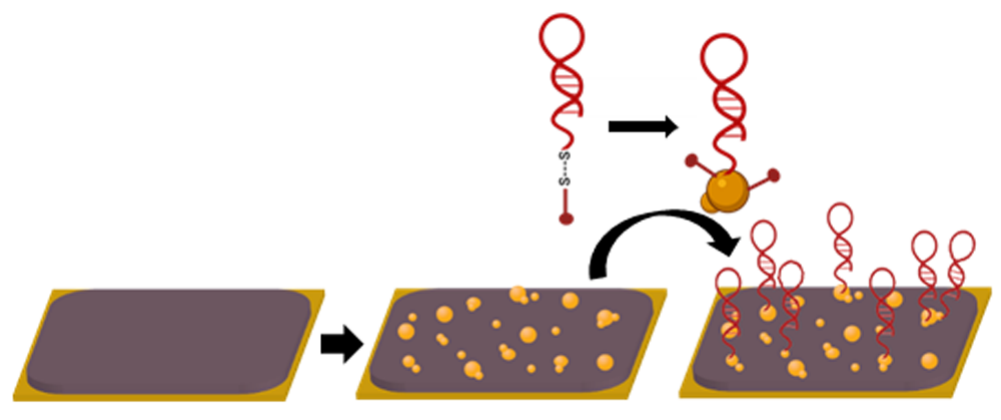

We demonstrate an organic electrochemical transistor (OECT) biosensor for the detection of interleukin 6 (IL6), an important biomarker associated with various pathological processes, including chronic inflammation, inflammaging, cancer, and severe COVID-19 infection. The biosensor is functionalized with oligonucleotide aptamers engineered to bind specifically IL6. We developed an easy functionalization strategy based on gold nanoparticles deposited onto a poly(3,4-ethylenedioxythiophene) doped with polystyrenesulfonate (PEDOT:PSS) gate electrode for the subsequent electrodeposition of thiolated aptamers. During this functionalization step, the reduction of sulfide bonds allows for simultaneous deposition of a blocking agent. A detection range from picomolar to nanomolar concentrations for IL6 was achieved, and the selectivity of the device was assessed against Tumor Necrosis Factor (TNF), another cytokine involved in the inflammatory processes.

## Introduction

Cytokines are bioactive proteins involved in proinflammatory and anti-inflammatory processes. Environmental stimuli, stress or diseases may trigger the release of these proteins in a ripple effect for the activation of the immune system and/or various metabolic pathways.^1,2^ Cytokine monitoring can thus be used to track progression of, e.g., infection, cancer, and acute or chronic inflammatory processes.^3−5^ Interleukin-6 (IL6) is a low molecular weight glycoprotein that belongs to the family of cytokines. IL6 has multiple functions, since it can act both as an anti-inflammatory protein, inhibiting other cytokines release, or in pro-inflammatory processes, triggering the acute phase response of the innate immune system.^6,7^ In physiological conditions and due to the local mode of action, IL6 is present in low concentrations (0.2–7.8 pg/mL; 10–300 fM)^8^ in human serum. In contrast, IL6 concentrations are much higher in severe sepsis (1652 pg/mL; 60 pM) or septic shock (8518 pg/mL; 320 pM)^9^ More recently, IL6 has also been associated with severe COVID-19 (SARS-CoV-2) infection. From preliminary studies, high levels (>3.5 pM)^10^ of IL6 were correlated with respiratory failure and death, making this cytokine an important prognostic marker for the disease and pandemic progression.^11^ For cytokine, in particular, IL6, detection and quantification represent a significant tool for precise monitoring of various diseases. Cytokines are present in fM-pM concentrations in bodily fluids; therefore, their detection requires both high sensitivity and selectivity. State of the art quantification methods include enzyme-linked immunosorbent assays (ELISA, Supporting Information, Table S1) and antibody arrays. Although these methods are robust and standardized, they require bulky equipment and laborious protocols, characteristics that hinder their usage at the point of care or even in at-home settings.^12^ For this reason, research has been focused on developing novel biosensor technologies for the rapid detection of these inflammatory biomarkers with miniaturized platform, such as electrochemical sensors^13−16^ or field effect transistors (FETs, Supporting Information, Table 2).^17−21^ Organic electrochemical transistors (OECTs) are now considered one of the most promising organic electronics technologies due to their high signal, low operation voltages, ease of fabrication, and ability to operate in aqueous environments.^22,23^ OECTs are three terminal devices, where the gate electrode modulates the current in the channel (source–drain current, *I*_*SD*_) by inflow or outflow of cations from an electrolyte solution into the poly(3,4-ethylenedioxythiophene) doped with polystyrenesulfonate (PEDOT:PSS) channel: small changes in gate voltage establish electrochemical modulation of the organic electronic channel, decreasing or increasing its conductivity, respectively.^24,25^ Via appropriate functionalization, OECTs can selectively detect the metabolite of interest through changes in *I*_SD_.^22,26^ OECTs have been used as sensing platform for numerous analytes, exploiting the possibility of modulating *I*_SD_ either through faradaic reactions at the gate or via nonfaradaic mechanisms that rely purely on the permeability of the semiconducting channel to ions from the electrolyte. The detection mechanism is usually achieved through specific enzymatic reactions,^27−35^ through molecularly imprinted polymers,^36,37^ or using nonfaradaic antibody–antigen recognition.^38−43^

Although antibodies remain the gold standard sensing units (e.g., in ELISA), there is a growing interest in finding innovative, more reproducible and robust solutions, based on protein or oligonucleotide scaffolds.^44^ Aptamers are oligonucleotide-based recognition elements which have been gaining interest due to their high selectivity, facility of synthesis, and low-cost development.^45^ These probes are generated through a technique called Systematic Evolution of Ligand by Exponential Enrichment (SELEX), based on selection and amplification of oligonucleotide sequences which bind target molecules.^46^ Aptamers were already employed successfully in electrochemical biosensors,^47−50^ immunoassays,^51−53^ and OECT-based biosensors.^54−56^

In this work, we present a planar OECT-based biosensor for the label-free detection of IL6 via aptamer recognition. The successful integration of aptamers in the sensor is attained by means of an easy functionalization method using Au nanoparticle (AuNPs) deposition and electrochemical aptamer grafting on the organic electronic gate electrode surface, while examples with a removable gold wire as gate exist,^17^ a planar geometry, and an easy functionalization strategy could aid in realizing a point-of-care device.

## Results and Discussion

**Figure 1:**
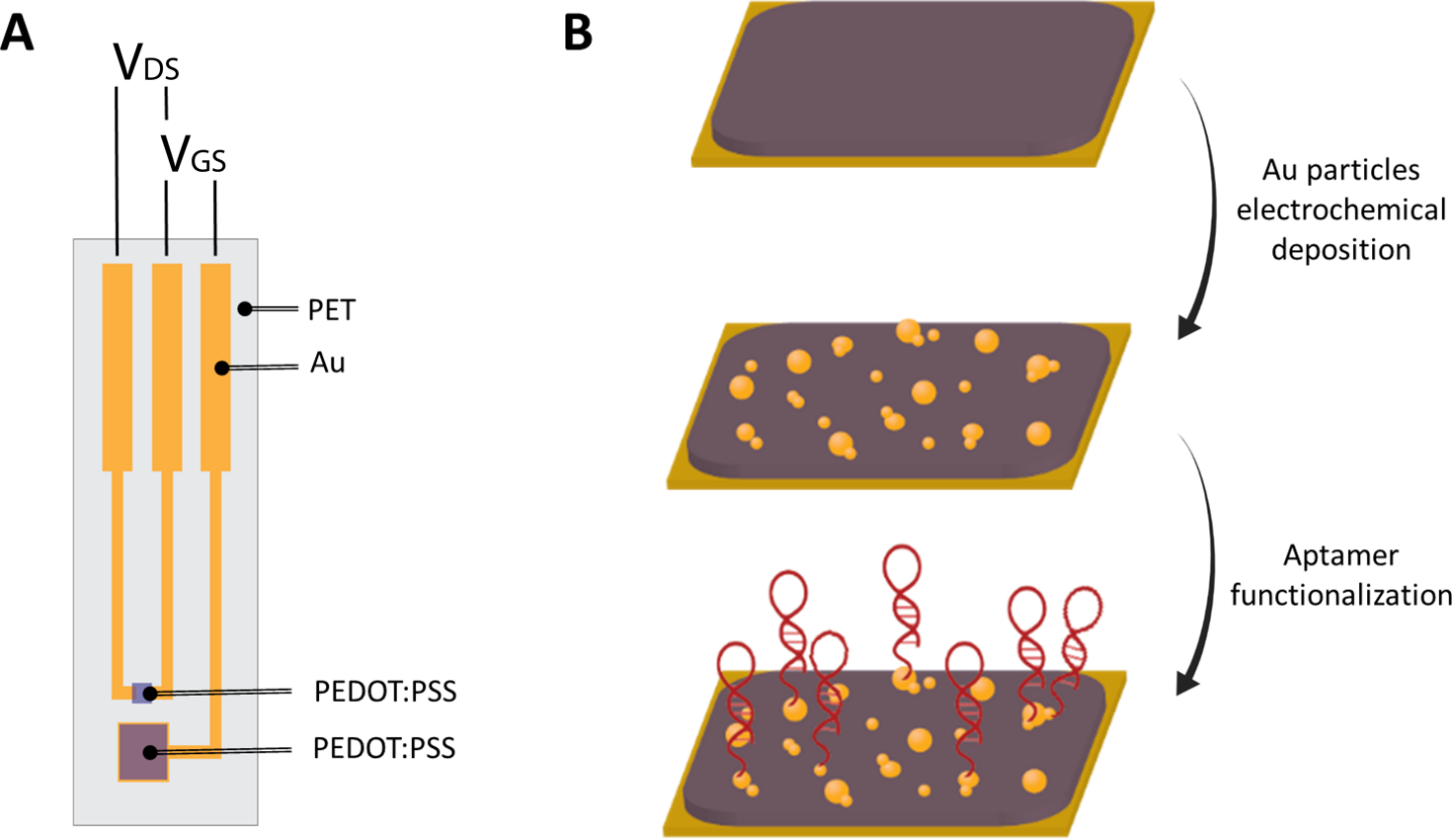
OECT-based aptasensor. (A) Schematic representation of the PEDOT:PSS based transistor planar configuration. Encapsulation (not shown) covers everything except the channel and the gate electrode. *V*_SD_ is the voltage between source and drain, and *V*_GS_ is the voltage applied between gate and source. (B) Gate functionalization steps: AuNP electrochemical deposition on PEDOT:PSS was followed by aptamer functionalization of the resulting composite.

OECTs were fabricated on a poly(ethylene naphthalate) (PEN) foil with standard photolithographic techniques and presented the gate electrode and the channel in a planar configuration (Figure 1A). Gold source, drain, and gate electrodes delineated the transistor geometry, and a thin layer of PEDOT:PSS constituted the channel. An additional layer of PEDOT:PSS was deposited on the active area of the gate electrode to increase its capacitance and to better modulate the channel at low applied voltages (Figure 1A).

**Figure 2:**
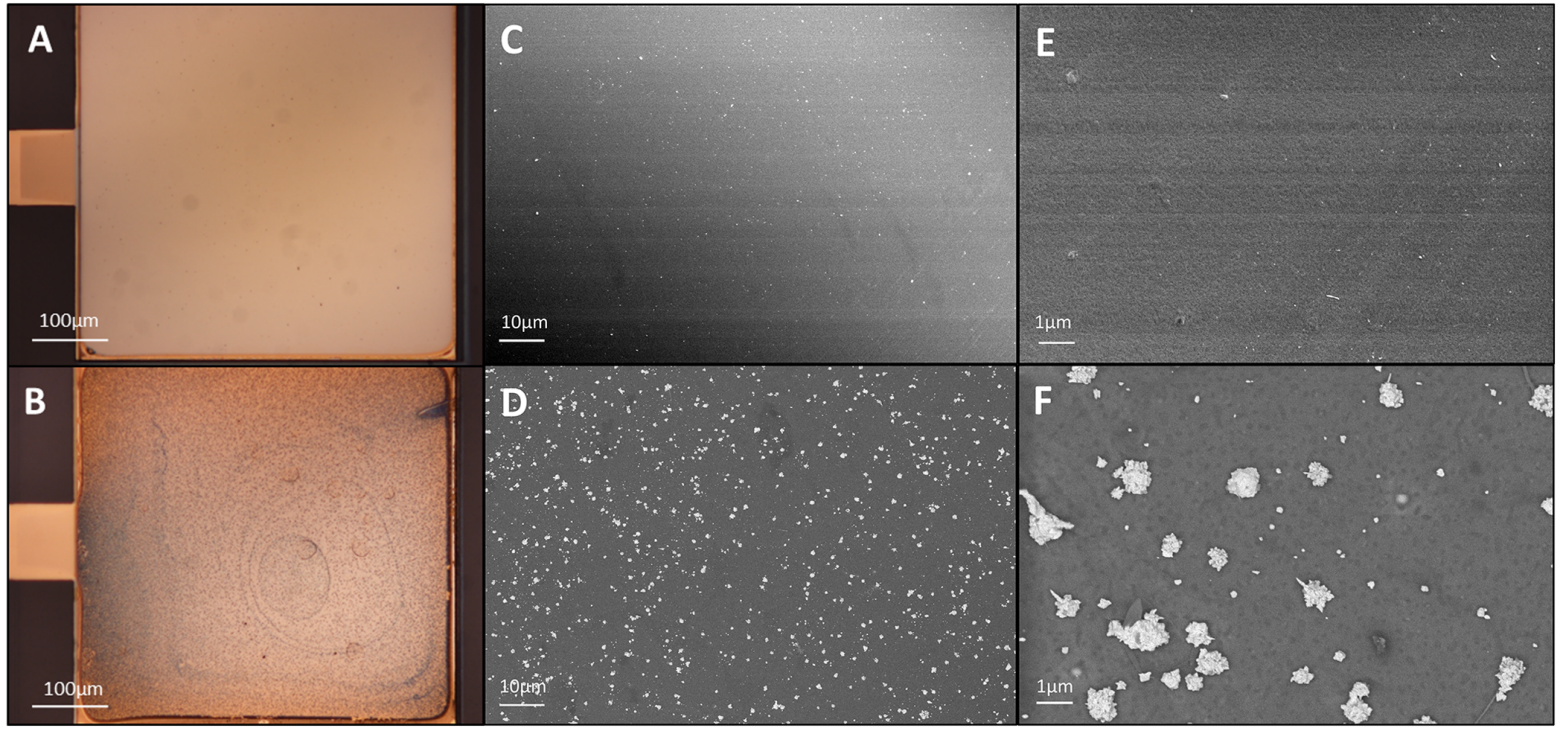
Modified gate electrode. Optical microscope images of the (A) PEDOT:PSS gate electrode and (B) AuNP/PEDOT:PSS. SEM images of (C, E) PEDOT:PSS and (D, F) the AuNP modified gate.

Conducting polymers and their composites have been widely used for analyte detection, even in cases in which they lack intrinsic specificity. Deposition of nanoparticles of different materials on polymer surfaces can provide additional physical-chemical properties, such as electrocatalytic activity using platinum nanoparticles (PtNPs) for peroxide detection^27,34^ or an easier surface for functionalization, such as AuNPs for thiolate sensing units.^57−60^ Here, the gate electrode was modified with AuNPs to provide a platform for aptamer binding. AuNP modification was performed via the electrochemical deposition of HAuCl_4_ on the PEDOT:PSS film (Figure 1B). The optical microscope images in Figure 2 show the presence of the AuNPs/aggregates (Figure 2B) when compared to that of a bare PEDOT:PSS gate electrode (Figure 2A). Through scanning electron microscopy (SEM), it was possible to study the disposition and dimension of the Au particles, visible as dispersed aggregates with submicron dimensions. Figure 2D and 2E clearly demonstrate the presence of AuNPs on the surface, when compared to the bare PEDOT:PSS device in Figure 2C and 2E. The presence of gold on top of the PEDOT:PSS layer was further demonstrated through cyclic voltammetry with ferricyanide [Fe(CN)_6_]^3–/4–^. The bare PEDOT:PSS electrode did not show electrochemical redox peaks from ferricyanide, while the AuNP/PEDOT:PSS surface did show characteristic peaks (Figure S1A). In contrast with a planar bare gold gate electrode with the same geometry, the PEDOT:PSS coated gate and AuNP/PEDOT:PSS showed better channel modulation, higher transconductance (Figure S1B), and faster transfer characteristic stabilization over time (Figure S1C).

**Figure 3:**
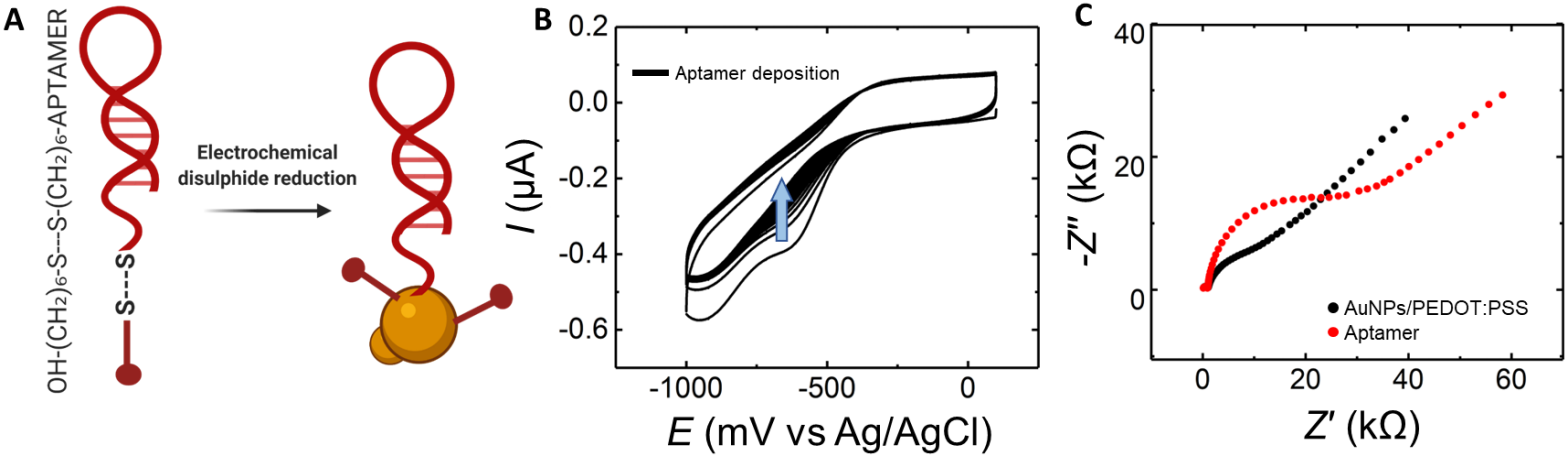
Aptamer functionalization. (A) Schematic representation of aptamer in solution and electrochemical disulfide reduction for AuNPs functionalization. (B) Cyclic voltammetry of AuNP/PEDOT:PSS gate electrode in 1 μM aptamer solution from 0 V to −1 V, 50 mV/s for 30 cycles. Trend indicated by arrow.(C) Impedance spectroscopy in ferricyanide 5 mM for AuNP/PEDOT:PSS electrode (black) and for the same electrode following aptamer immobilization (red).

The specific aptamer sequence selective for IL6 was developed by Rhinehardt et al, through SELEX, and evaluated with molecular dynamics analysis and Surface Plasmon Raman imaging (SPRi).^61^ Furthermore, the sequence has already been proved applicable for IL6 biosensing technology based on Surface-Enhanced Raman Spectroscopy (SERS).^62^ Functionalization of AuNPs with aptamer was performed via electrochemical disulfide reduction at the gate electrode. In solution, aptamers are present in their stabilized form, with the thiolate ending protected through a 6-mercapto-1-hexanol (MCH) residue (Figure 3A). MCH is a molecule often used in combination with aptamers to create an antifouling layer on the sensing surface.^63,64^ We took advantage of the aptamer-MCH configuration, performing an electrochemical reduction at the gate electrode, binding both aptamer and linker to the gold particles. Figure 3B shows cyclic voltammetry of a AuNP/PEDOT:PSS gate electrode in an aptamer solution. We observed a decrease of cathodic current over time and the decrease of the reduction peak around −600 mV vs Ag/AgCl, indicating the binding of aptamer and MCH to the electrode. To prove the presence of the aptamer on the AuNP/PEDOT:PSS surface, we performed faradaic electrochemical impedance spectroscopy (EIS) analysis on the gate electrode before and after aptamer deposition (Figure 3C). The binding of the aptamer and MCH to the AuNPs on the gate electrode created a barrier to the negatively charged redox molecule and consequently an increase in the charge transfer resistance (*R*_*ct*_), visible as a widening of the semicircular component of the trace toward larger *Z*′.

**Figure 4:**
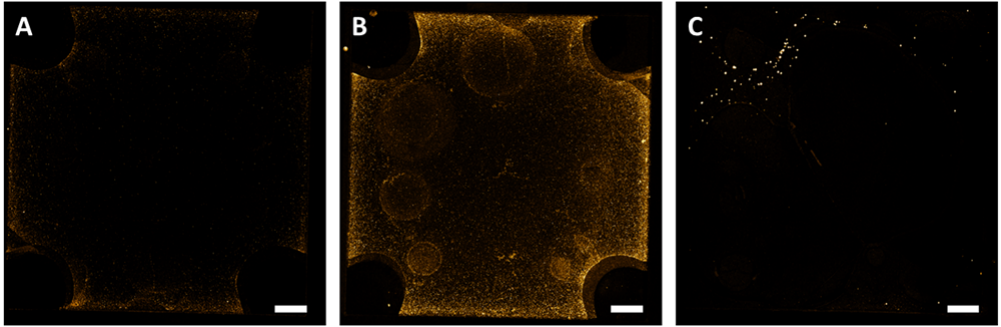
Fluorescent oligonucleotide functionalization. (A) Fluorescent image of AuNP/PEDOT:PSS (no Cy3-tag). (B) Fluorescence image with a Cy3 tag on the 3′-end (Cy3-aptamer/AuNP/PEDOT:PSS). (C) Cy3-aptamer on a gate without AuNP (Cy3-aptamer/PEDOT:PSS). Scale bar: 50 μm

By using a fluorescent tag (Cy3) at the 3′-end of the sequence, we could obtain confocal fluorescent images of the functionalized gate (Figure 4). In Figure 4B, the fluorescent image of a fully functionalized gate with aptamers and a Cy3 tag is shown. Compared with a gate with only AuNP/PEDOT:PSS (Figure 4A), the gate area with fluorescent tagged oligonucleotides is evident. The control of fluorescent tagged aptamers on PEDOT:PSS without AuNP is shown in Figure 4C. While fluorescent spots are also visible without AuNP, the coverage is much lower. The functionalized gate surface was further studied using Fourier-transform infrared spectroscopy (FTIR) in attenuated total reflection (ATR) mode (Figure S2). FTIR-ATR confirms the presence of oligonucleotides (aptamers) bound to the AuNP/PEDOT:PSS surface. The PEDOT:PSS peaks are stronger than the aptamer ones due to the greater abundance of the polymer compared to the DNA monolayer on the gold particles. In Figure S2 it is possible to notice visible changes from the AuNP/PEDOT:PSS surface (orange spectra) to the Aptamer/AuNP/PEDOT:PSS functionalized electrode (purple spectra). Specifically, there is an increase in absorption at 1100 cm^–1^ (phosphate groups), along with weaker increases at 1230 cm^–1^ (amine and phosphate groups) and 1600–1700 cm^–1^ (amine and carbonyl groups). These changes in the spectra overlap in the absorption region with a reference electrode, which exhibits just bare gold and aptamer on the surface (violet spectra).

**Figure 5:**
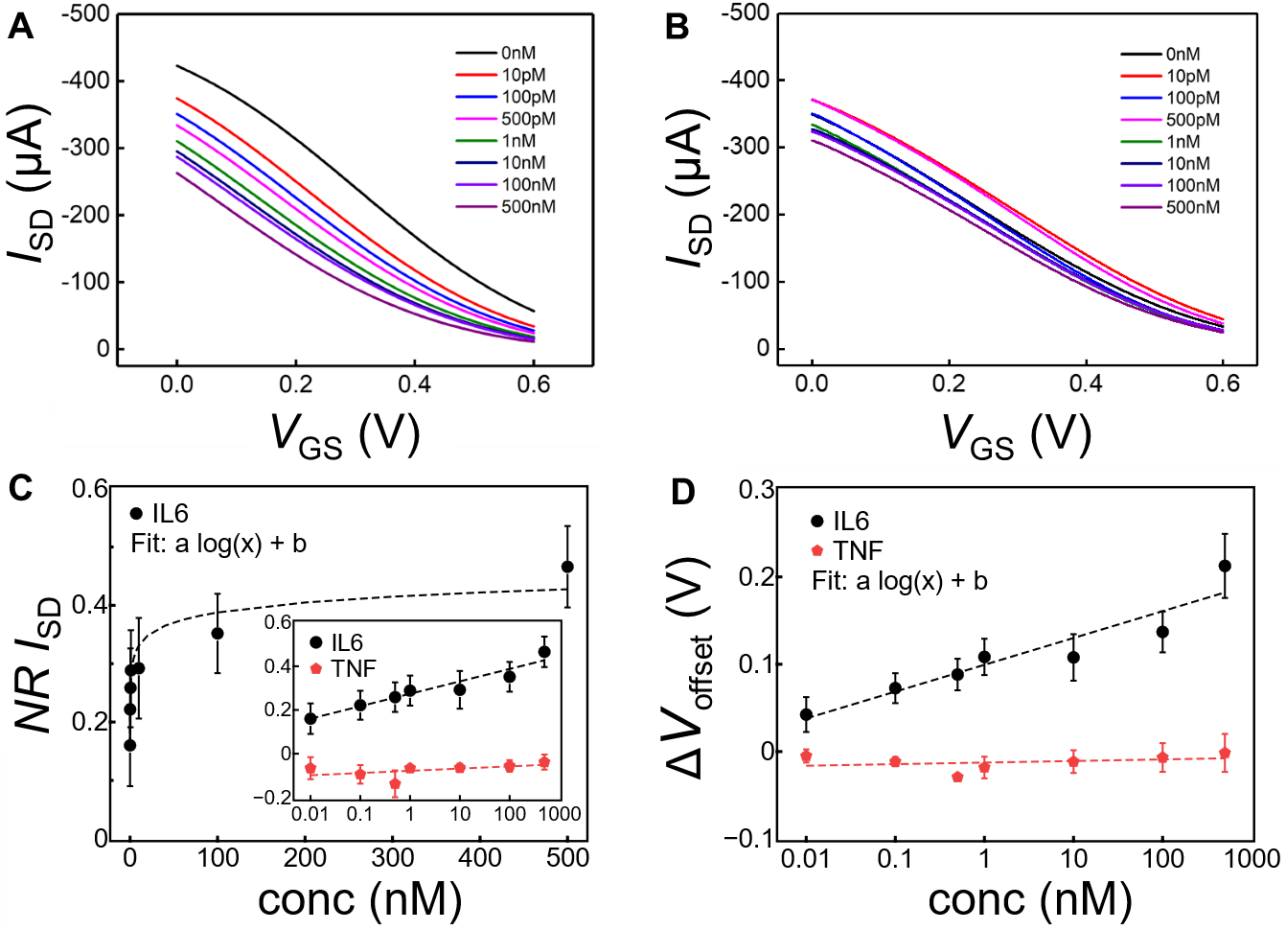
Interleukin-6 sensing. (A) Transfer characteristics of an OECT based aptasensor for increasing the concentration of IL6 in Tween buffer, recorded at fixed *V*_DS_ −0.3 V and sweeping *V*_GS_ from 0 to 0.6 V. (B) Transfer characteristics of OECT based aptasensor for increasing concentration of TNF in Tween 20 buffer, recorded at fixed *V*_DS_ −0.3 V and sweeping *V*_GS_ from 0 to 0.6 V. (C) Normalized response (NR_ISD_) obtained for *V*_GS_ 0.3 V upon increasing concentration of IL6 (black dots, fit: *a* = 2.5 × 10^–2^ and *b* = 0.3; *n* = 5).The inset shows the lin/log graph with the dotted lines as an eye-guide with for IL6 (same as for lin/log plot), and for TNF (with parameters *a* = 4.4 × 10^–3^ and *b* = −8 × 10^–2^; *n* = 4). (D) Gate voltage variations obtain for increasing concentration of IL6 (black dots, fit: *a* = 1.3 × 10^–2^ and *b* = 0.1; *n* = 5) and TNF (red dots, fit: *a* = 8 × 10^–4^ and *b* = −1.3 × 10^–2^; *n* = 4).

We monitored the OECT response for different concentrations of IL6, sweeping the voltage between source and gate (*V*_*GS*_) from 0.0 to 0.6 V and keeping the voltage between source and drain (*V*_*DS*_) fixed at −0.3 V (Figure 5A). The measurements were performed in phosphate buffer plus 0.05% Tween-20 (polysorbate 20), containing increasing IL6 concentrations from 10 pM to 500 nM. Tween-20 is a surfactant commonly used in standard techniques like ELISA to reduce unspecific binding.^65^ Here, we diluted the analyte of interest in solutions containing Tween-20 to prevent possible protein adsorption on either PEDOT:PSS or uncovered gold surfaces. Figure 5A shows an overlay of the transfer characteristics for increasing concentrations of IL6. The curves are characterized by the monotonic decrease of *I*_*SD*_ proportional to [IL6] when the gate is functionalized with aptamer. In order to quantify the response of the biosensor, we constructed the dose curve calculating the normalized response (*NR*), for the IL6 analyte (Figure 5C) and for a control molecule TNF (Tumor Necrosis Factor), an analogous cytokine with different structure but similar function. Increasing concentrations of TNF induced much smaller current changes (Figure 5B) that, moreover, are not monotonic with [IL6] with *NR*_*I*_SD__ consistently around −0.1 (inset Figure 5C). Similarly, an OECT without aptamer functionalization showed no monotonic behavior with [IL6] (Figure S3). Owing to the logarithmic variation of *NR* vs [IL6], we immediately understand that the sensitivity of this device is greatly enhanced at the smallest concentrations, as d*NR*/d*c* scales as 1/*c*, with *c* being the concentration. This sublinear response of our device is key to imparting a greater sensitivity at the lowest concentrations, which are the physio-pathological relevant in the case of IL6. A limit of detection (LOD) of about 24 pM was extracted as (*m* + 3δ) using the TNF measurements as control solution (from four devices). In the formula, *m* is the average current value of the control solution and δ its standard deviation.

The working mechanism of p-type depletion mode OECT devices relies on the channel dedoping by cation inflow, accompanied by the ion migration from the electrolyte, upon application of increasing *V*_GS_. The drain current *I*_SD_ depends on applied voltages and intrinsic material properties, and can be described following the model proposed by Bernards and Malliaras:^26^
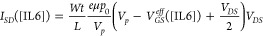
1Here *W*, *t*, and *L* are the channel width, thickness, and length, *e* is the elementary charge, μ is the hole mobility in the channel, *p*_0_ is the initial hole density in the semiconductor, *V*_*p*_ is the pinchoff voltage, and *V*_*GS*_^*eff*^ is the effective gate voltage. The latter depends on a voltage offset *V*_offset_ directly proportional to the concentration of the analyte when the gate electrode is functionalized with a specific biorecognition group, and the device is operated as a biosensor (Figure 5D):
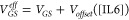
2

The offset voltage represents a potential variation at gate/electrolyte interface,^26,66^ induced by the binding between IL6 and aptamer, which consequently determines a current variation in the channel. As shown in Figure 5D, increasing concentration of IL6 produces an offset voltage variation (black dots, number of devices *n* = 5), absent in the case of the control cytokine TNF (red dots, *n* = 4). The corresponding logarithmic fit hints that the response is due to the contribution of the concentration to the potential drop at the gate/electrolyte interface. We further investigated the electrical characteristics, studying the changes in transconductance *g*_m_ (slope of the transfer curve) for increasing concentration of analytes (Figures S4A and S4B). We calculated the normalized response for the peak of *g*_m_ (*NRg*_m_) (Figure S4C). Further we extracted the *V*_GS_ at which devices show the maximum transconductance, comparing the relative changes between 0 M and increasing analyte concentrations (*NRV*_GS_). While the normalized response of *g*_m_ varied following both [IL6] and [TNF], implying that Δ*g*_m_ may have a contribution from the device stress itself (Figure S4C), the normalized response for (*V*_GS_) at maximum transconductance is more selective toward IL6 concentrations (Figure S4D). The *V*_GS_ shows a decreasing behavior, upon increasing IL6 concentration, in contrast with the static TNF behavior. We hypothesized that this potential change (*V*_GS_) may be attributed to conformational aptamer adjustments upon IL6 binding, which could affect the charge density closer to the electrode surface, thus modifying the interface potential. EIS measurements for IL6 can be seen in Figure S5A. For comparison with the OECT sensor the highest normalized response was at 1 Hz and is compared to the OECT response in Figure S5B. It indicates that for concentrations lower than 100 nM the OECT sensor exhibits a higher response than EIS measurements.

## Conclusion

In this work, we showed an aptamer-based OECT biosensor for protein recognition. The sensor quantifies the concentration of the analyte of interest in pathological ranges against another analogous protein. The OECT shows changes in electrical characteristics such as *I*_SD_ current and *V*_GS_ for the maximum *g*_m_, providing information on the binding between aptamer and protein. Moreover, we show an easy functionalization strategy based on electrochemical AuNP deposition and electrochemical reduction of the sensing unit at the gate electrode. This electrochemical reduction step of the sensing unit allows for not only targeted deposition on the aptamer but also a simultaneous deposition of a blocking agent, further simplifying the fabrication process. In particular, when envisioning a portable sensing platform to detect multiple analytes, this strategy is advantageous compared to chemically reducing the thiol bond of the sensing unit. These strategies, together with the fabrication steps, make the device disposable and functional for a point of care application, paving the way for easy health monitoring. Further advancement will foresee integration with microfluidics and multiple detection of cytokines on different OECT, to allow a more complete understanding of the inflammatory condition of the patient.

## Materials and Methods

### Device Fabrication

A circular 4 in. substrate was obtained by cutting poly(ethylene naphthalate) (PEN) foil (Teonex Q65HA, 125 μm, Peutz Folien GmbH). After the PEN foil was cleaned with water and acetone, 2 nm chromium (Cr) and 50 nm gold (Au) were thermally evaporated onto the surface. Gold contacts, wirings and gates were patterned using a Shipley 1805 positive resist and photolithography (Karl Suss MA/BM 6 mask aligner), then wet etched in I_2_/KI solution for Au, and H_2_O_2_/NH_4_Cl/H_2_O for Cr. The substrate was then stripped with acetone. Channels and gates were deposited by spin coating (1400 rpm for 30s) with a PEDOT:PSS (Clevios PH1000) solution with 5% v/v ethylene glycol and 1% v/v GOPS (3-glycidyloxypropyl)trimethoxysilane) and dodecylbenzenesulfonic acid (1 drop per 5 mL) and patterned using a Shipley 1813 positive resist, after baking at 120 °C for 2h. The substrate was then dry reactive ion etched with CF_4_/O_2_ and stripped with acetone. The channel has a geometry of 20 μm × 100 μm and the gate of 250 μm × 250 μm. As a final step, the substrate was encapsulated with SU-8 2010 (MicroChem) and openings were defined by using wet etching with developer mr-Dev 600 (Microresist Techonology). Chemicals were used as received from Sigma-Aldrich unless stated otherwise.

### Gate Functionalization

Gold nanoparticles were formed by applying −0.273 V vs Ag/AgCl in 3 mM HAuCl_4_ in 0.1 M KCl for 60 s through electrochemical deposition (CH Instruments potentiostat 760c model with a three-electrode setup) at the gate electrode. Devices were then rinsed thoroughly with DI water. The aptamer sequence (5′-CTT CCA ACG CTC GTA TTG TCA GTC TTT AGT-3′) was found in the literature.^61^ This sequence, with a thiolated 6-mercapto-1-hexanol group (MCH) at the 5′ end, was purchased from Sigma-Aldrich. A solution of 1 μM aptamer in 10 mM phosphate buffered saline (PBS) was used for electrochemical deposition of the oligomer on the gold particles, using cyclic voltammetry for 30 cycles from 0.1 to −1 V at 50 mV/s on the gate electrode. After DI water rinsing, the surface electrode was rearranged in a solution of KCl 50 mM for 20 cycles from −0.1 to 0.1 V at 50 mV/s. The active area of the device was then incubated in 0.05% Tween 20 buffer in 10 mM PBS for 1 h. Gold nanoparticle deposition was observed performing cyclic voltammetry in 5 mM ferricyanide solution at the gate electrode from −0.5 to 0.5 V at 50 mV/s for 2 cycles. AuNP deposition was also studied with optical and scanning electron microscopy (SEM). Aptamer deposition was observed through impedance measurements in 5 mM ferricyanide and with Fourier-transform infrared spectroscopy in attenuated total reflectance mode (FTIR-ATR) by using a Bruker Equinox 55 spectrometer equipped with a Bruker Platinum ATR single reflection accessory. The spectra were accumulated over 50 scans in the range 4000 to 500 cm^–1^ with a resolution of 4 cm^–1^ using a zero-filling factor of 2. The background measurement was PEN with a thermally evaporated Au layer, like the substrate used for device preparation. (Additional corrections, such as baseline adjustments, have not been applied to the measured data.) Fluorescent microscopy images were taken with a Zeiss LSM 980, Airyscan 2 (Carl Zeiss AG). The sequence for the fluorescent image was 5′-ThioMC6-D/AAA AAA TTA CCG GGC TCT GCC ATC T/3Cy-3′ with a thiol group at the 5′ end and a Cy3-tag at the 3′ end.

### Electrical Characterization

Electrical measurements were performed in 10 mM PBS buffer with or without 0.05% Tween 20. Buffer with Tween 20 was used in experiments for sensing IL-6. Source, gate, and drain electrodes were connected to a Keithley 2600 source meter, and all measurements were carried out at room temperature. The transfer characteristics were performed by keeping the voltage between source and drain fixed at *V*_DS_=-0.3 V and sweeping the voltage between source and gate *V*_GS_ from 0 to 0.6 V. The OECT was incubated with increasing concentration of IL6 or TNF in Tween 20 buffer for 15 min and then recorded until current stabilization (no variation in the CV noticeable anymore, approximately 10 min). Au, PEDOT:PSS, and AuNP/PEDOT:PSS electrodes were stabilized by sweeping gate voltage from 0 to 0.6 V and keeping the source–drain voltage fixed at −0.3 V for 15 min.

### Data Analysis

To quantify and compare different devices, a normalized *I*_SD_ response (*NR*_*I*_SD__) was defined as
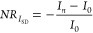
3where *I*_*n*_ is the current taken at *V*_GS_ = 0.3 V for each n^th^ concentration of IL6 and TNF, and *I*_0_ is the baseline current at *V*_GS_ = 0.3 V for the solution at 0 M analyte concentration. The normalized *g*_m_ response was similarly calculated as
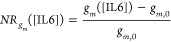
4where *g*_*m*_ ([IL6]) is the transconductance peak value at concentration [IL6] (and similarly for TNF) and *g*_*m*,0_ is the transconductance for the solution at 0 M analyte concentration. The sensor’s capability was also investigated by comparing relative changes at the *V*_GS_ for the maximum *g*_m_ using
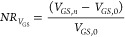
5where *V*_*GS*_ ([IL6]) is the voltage between gate and source for maximum *g*_m_ taken for each concentration of IL6 (and similarly with TNF) and *V*_*GS*,0_ is the voltage between source and gate for maximum *g*_m_ for the solution at 0 M analyte concentration.

## Data Availability

Experimental data are available on Zenodo.^67^

## Supporting Information Available

Table S1, ELISA kits for IL-6 detection; Table S2, cytokine sensors with electrolyte gated organic transistors; Figure S1, AuNP/PEDOT:PSS electrical characteristics; Figure S2, FTIR-ATR measurements; Figure S3, IL6 detection on AuNP/PEDOT:PSS gate; Figure S4, transconductance of OECT based aptasensor; and Figure S5, comparison of OECT and electrochemical impedance spectroscopy sensor responses (PDF)
