# Supplementary material for: Organic Electrochemical Transistor Aptasensor for Interleukin-6 Detection
Source: ACS Appl Mater Interfaces. 2023 Dec 23;16(45):61467–74. doi: 10.1021/acsami.3c12397 (PMC11565573; doi:10.1021/acsami.3c12397)
Supplement: Supplementary file 1 — am3c12397_si_001.pdf [file am3c12397_si_001.pdf]

Supporting information

Organic electrochemical transistor aptasensor for interleukin-6 detection

Chiara Diacci[1,2], Bernhard Burtscher[1], Marcello Berto[2], Tero-Petri Ruoko[1], Samuel Lienemann[1], Pierpaolo Greco[3,4], Magnus Berggren[1], Marco Borsari[5], Daniel T. Simon[1*], Carlo A. Bortolotti[2*], Fabio Biscarini[2,4]

1. Laboratory of Organic Electronics, Department of Science and Technology, ITN, Linköping University, 601 74, Norrköping, Sweden.
2. Dipartimento di Scienze della Vita, Università di Modena e Reggio Emilia, via Campi 103, 41125 Modena, Italy.
3. Department of Neuroscience and Rehabilitation, Università di Ferrara, Via Borsari 46, 44121 Ferrara, Italy.
4. Center for Translational Neurophysiology of Speech and Communication, Istituto Italiano di Tecnologia, via Fossato di Mortara 17-193, 44100 Ferrara, Italy.
5. Dipartimento di Scienze Chimiche e Geologiche, Università di Modena e Reggio Emilia, via Campi 103, 41125 Modena, Italy.

Corresponding authors: daniel.simon@liu.se, carloaugusto.bortolotti@unimore.it

Table S1. State of the art ELISA kits for IL-6 detection. Numbers are from the latest kits available on the websites (accessed, 18th October 2023).

IL-6	LSBio	Sensitivity: 14.2 fM (0.3pg/ml); Range: 4.69-300 pg/ml
	Invitrogen	Sensitivity: 2fM – 100 fM (0.06-2 pg/ml) depending on the kit
	R&D system	Sensitivity: 33fM (0.7 pg/ml); Range: 3.1-300 pg/ml

Table S2. Cytokine sensors with electrolyte gated organic transistors.

Target molecule	Sensing unit	Channel material	Reported LoD	Ref.
**Interleukin 6 (IL6)**	Antibody	PEDOT:PSS	-	[1,2]
**Interleukin 4 (IL-4)**	Antibody	Pentacene	5 nM	[3]
**Interleukin 6 (IL-6)**	Antibody/aptamer	Pentacene	- / 1 pM	[2,4]
**Tumor necrosis factor-*α* (TNF-*α*)**	Antibody	Pentacene	-	[5]
**Tumor necrosis factor-*α* (TNF-*α*)**	Affimer	Pentacene	1 pM	[6]
**C-reactive protein (CRP)**	Antibody	P3HT	2 pM / 590zM / -	[7–9]

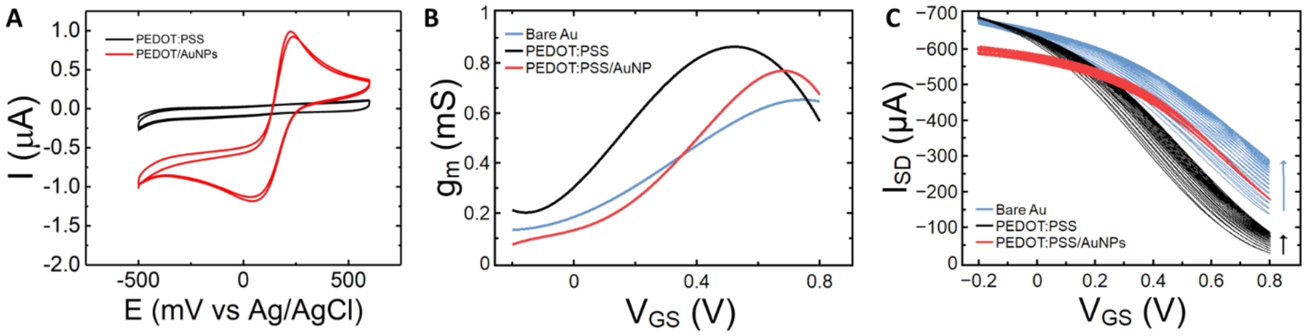

Figure S1.AuNP/PEDOT:PSS electrical characteristics. (A) Cyclic voltammetry in 5 mM ferricyanide from -0.5 V to 0.6 V at 50 mV/s for PEDOT:PSS (black) and AuNP/PEDOT:PSS (red) gate electrode. (B) Transconductance g_m for bare Au (blue), PEDOT:PSS (black), and AuNP/PEDOT:PSS (red). (C) Transfer characteristics for bare Au (blue), PEDOT:PSS (black), and AuNP/PEDOT:PSS (red) gate electrodes modulating the same channel for 15 minutes. Transfer curves were obtained sweeping V_{GS} back and forward from -0.2 V to 0.8 V and keeping V_{DS} fixed at -0.3 V.

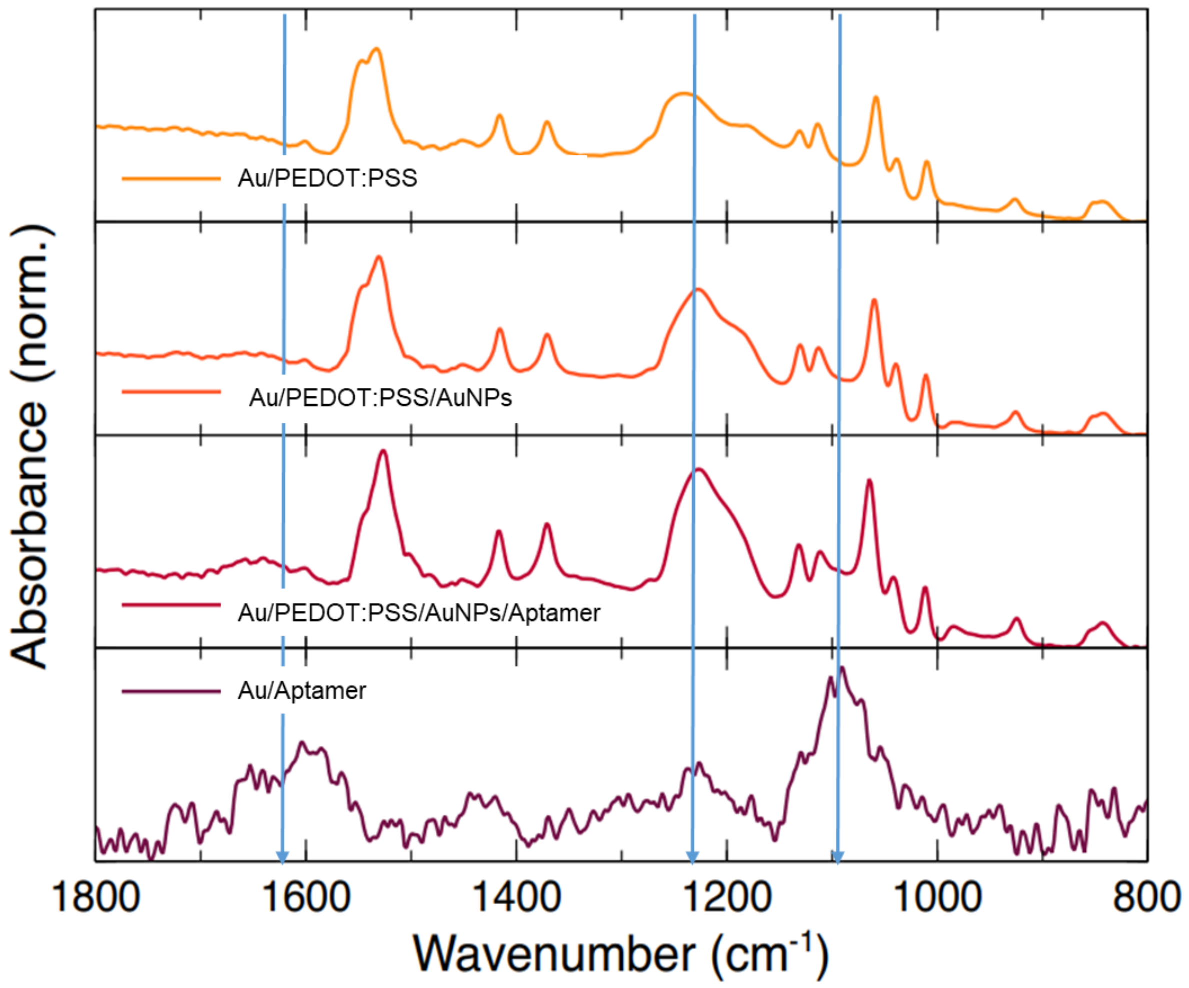

Figure S2. FTIR-ATR measurements. FTIR-ATR spectra for PEDOT:PSS electrode (yellow), AuNP/PEDOT:PSS electrode (orange), aptamer/AuNP/PEDOT:PSS electrode (purple), and aptamer/Au electrode (violet).

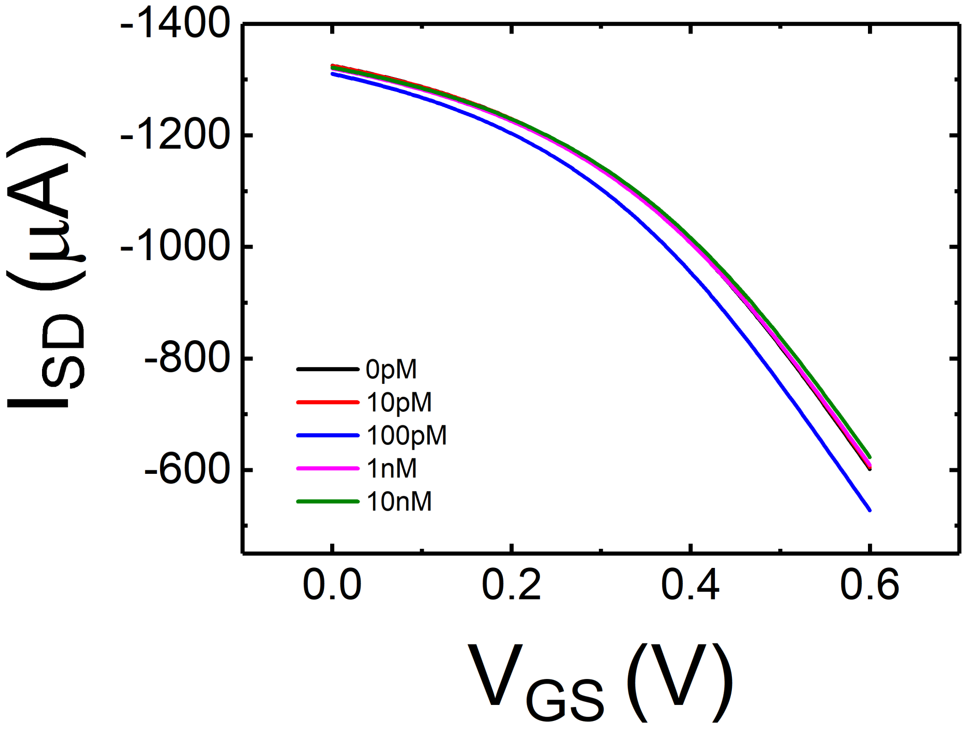

Figure S3. IL6 detection on AuNP/PEDOT:PSS gate. Transfer characteristics of the AuNP/PEDOT:PSS-only (i.e., no aptamer) OECT for increasing concentration of IL6 in PBS buffer, recorded at fixed V_{DS} = -0.3 V and sweeping V_{GS} from 0 V to 0.6 V.

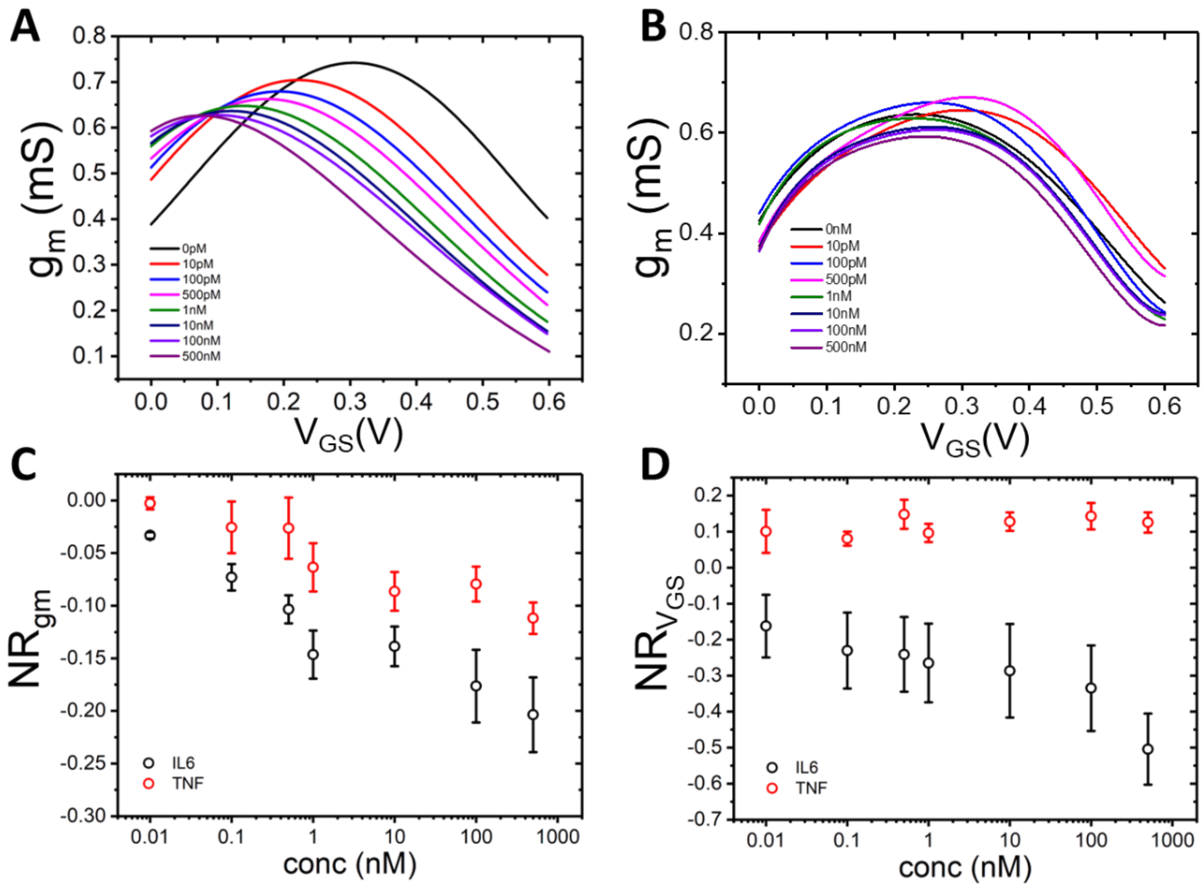

Figure S4. Transconductance of OECT based aptasensor. Change of g_m for increasing concentration of (A) IL6 and (B) TNF in Tween 20 buffer, obtained from transfer curves recorded at fixed V_{DS} = -0.3V and sweeping V_{GS} from 0 V to 0.6 V. (C) Normalized transconductance response as a function of [IL6] (black circles) and [TNF] (red circles). (D) V_{GS} normalized response obtain from V_{GS} at the maximum of transconductance for IL6 (black circles) and TNF (red circles). Error bars represent the standard error for five IL6 measurements (black) and four TNF measurements (red) on different devices.

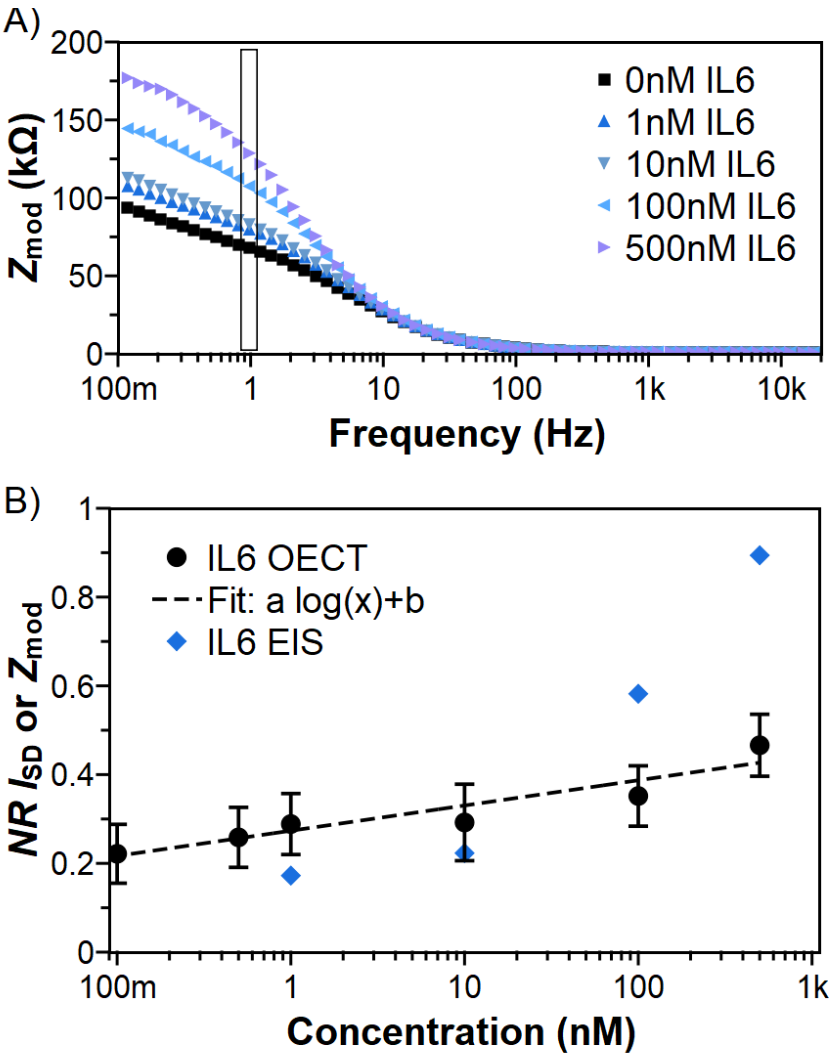

Figure S5 Electrochemical impedance spectroscopy aptasensor response to analyte and comparison to OECT aptasensor. EIS recorded in 5 mM ferricyanide vs 170mV for aptamer functionalized AuNP/PEDOT:PSS gate electrode as function of increasing concentrations of IL6 in Tween 20 buffer (A). Normalized response of Z_{mod} at 1Hz for IL6 compared to OECT response(B).

Supplementary References:

(1) Gentili, D.; D'Angelo, P.; Militano, F.; Mazzei, R.; Poerio, T.; Brucale, M.; Tarabella, G.; Bonetti, S.; Marasso, S. L.; Cocuzza, M.; Giorno, L.; Iannotta, S.; Cavallini, M. Integration of Organic Electrochemical Transistors and Immuno-Affinity Membranes for Label-Free Detection of Interleukin-6 in the Physiological Concentration Range through Antibody-Antigen Recognition. *J. Mater. Chem. B* **2018**, *6* (33), 5400–5406. https://doi.org/10.1039/c8tb01697f.

(2) Manco Urbina, P. A.; Berto, M.; Greco, P.; Sensi, M.; Borghi, S.; Borsari, M.; Bortolotti, C. A.; Biscarini, F. Physical Insights from the Frumkin Isotherm Applied to Electrolyte Gated Organic Transistors as Protein Biosensors. *J. Mater. Chem. C* **2021**, *9* (33), 10965–10974. https://doi.org/10.1039/D1TC02546E.

(3) Casalini, S.; Dumitru, A. C.; Leonardi, F.; Bortolotti, C. A.; Herruzo, E. T.; Campana, A.; De Oliveira, R. F.; Cramer, T.; Garcia, R.; Biscarini, F. Multiscale Sensing of Antibody-Antigen Interactions by Organic Transistors and Single-Molecule Force Spectroscopy. *ACS Nano* **2015**, *9* (5), 5051–5062. https://doi.org/10.1021/acsnano.5b00136.

(4) Diacci, C.; Berto, M.; Di Lauro, M.; Bianchini, E.; Pinti, M.; Simon, D. T.; Biscarini, F.; Bortolotti, C. A. Label-Free Detection of Interleukin-6 Using Electrolyte Gated Organic Field Effect Transistors. *Biointerphases* **2017**, *12* (5), 05F401. https://doi.org/10.1116/1.4997760.

(5) Berto, M.; Casalini, S.; Di Lauro, M.; Marasso, S. L.; Cocuzza, M.; Perrone, D.; Pinti, M.; Cossarizza, A.; Pirri, C. F.; Simon, D. T.; Berggren, M.; Zerbetto, F.; Bortolotti, C. A.; Biscarini, F. Biorecognition in Organic Field Effect Transistors Biosensors: The Role of the Density of States of the Organic Semiconductor. *Anal. Chem.* **2016**, *88* (24), 12330–12338. https://doi.org/10.1021/acs.analchem.6b03522.

(6) Berto, M.; Diacci, C.; D'Agata, R.; Pinti, M.; Bianchini, E.; Lauro, M. Di; Casalini, S.; Cossarizza, A.; Berggren, M.; Simon, D.; Spoto, G.; Biscarini, F.; Bortolotti, C. A. EGOFET Peptide Aptasensor for Label-Free Detection of Inflammatory Cytokines in Complex Fluids. *Adv. Biosyst.* **2018**, *2* (2), 1–8. https://doi.org/10.1002/adbi.201700072.

(7) Magliulo, M.; De Tullio, D.; Vikholm-Lundin, I.; Albers, W. M.; Munter, T.; Manoli, K.; Palazzo, G.; Torsi, L. Label-Free C-Reactive Protein Electronic Detection with an Electrolyte-Gated Organic Field-Effect Transistor-Based Immunosensor. *Anal. Bioanal. Chem.* **2016**, *408* (15), 3943–3952. https://doi.org/10.1007/s00216-016-9502-3.

(8) Macchia, E.; Manoli, K.; Holzer, B.; Di Franco, C.; Picca, R. A.; Cioffi, N.; Scamarcio, G.; Palazzo, G.; Torsi, L. Selective Single-Molecule Analytical Detection of C-Reactive Protein in Saliva with an Organic Transistor. *Anal. Bioanal. Chem.* **2019**, *411* (19), 4899–4908. https://doi.org/10.1007/s00216-019-01778-2.

(9) Palazzo, G.; De Tullio, D.; Magliulo, M.; Mallardi, A.; Intranuovo, F.; Mulla, M. Y.; Favia, P.; Vikholm-Lundin, I.; Torsi, L. Detection Beyond Debye's Length with an Electrolyte-Gated Organic Field-Effect Transistor. *Adv. Mater.* **2015**, *27* (5), 911–916. https://doi.org/10.1002/adma.201403541.
